# Determination of susceptibility to sensitization to dental materials in atopic and non-atopic patients

**DOI:** 10.4317/medoral.17424

**Published:** 2011-12-06

**Authors:** Gonzalo Rojas-Alcayaga, Alonso Carrasco-Labra, Paula Danús, María A. Guzmán, Irene Morales-Bozo, Blanca Urzúa, Ana Ortega-Pinto

**Affiliations:** 1 Department of Pathology, Faculty of Dentistry, University of Chile, Dento-Maxilo-Facial Service, Clinical Hospital University of Chile, Sergio Livingstone 943, Independencia. Santiago-Chile; 2 Department of Oral and Maxillofacial Surgery, Faculty of Dentistry, University of Chile. Evidence-Based Dentistry Unit, Faculty of Dentistry, University of Chile, Sergio Livingstone 943, Independencia. Santiago-Chile; 3 Dentistry, Currently Private Practice, Sergio Livingstone 943, Independencia, Santiago-Chile; 4 Allergy Center, Clinical Hospital University of Chile, Santos Dumont 999, Independencia, Santiago-Chile; 5 Department of Basic and Communitarian Sciences, Faculty of Dentistry, University of Chile, Sergio Livingstone 943. Independencia. Santiago-Chile; 6 Department of Basic and Communitarian Sciences, Faculty of Dentistry, University of Chile, Sergio Livingstone 943. Independencia. Santiago-Chile; 7 Department of Pathology, Faculty of Dentistry, University of Chile, Sergio Livingstone 943, Independencia. Santiago-Chile

## Abstract

Introduction: Some studies report that atopic patients have a greater frequency of delayed-type sensitization than non-atopic patients. Objective: To determine the influence of the atopic condition on delayed sensitization to dental materials. Design: cross-sectional study. Methods: Forty (40) atopic subjects and forty (40) non-atopic subjects, of both sexes, between 20 and 65 years of age were included. The determination of delayed sensitization to dental materials was performed using patch test. An oral exam was also carried out to check for lesions of the oral mucosa. Results: 61.25% of the patients were positive for delayed-type sensitization to one or more allergens, being palladium chloride (21.25%), ammoniated mercury (20%), benzoyl peroxide (12.5%) and amalgam (10%) the most frequent. The frequency of sensitization was 67.5% in the group of atopic patients, compared to 55% in the non atopic group (p>0.05). The materials with the greatest difference of sensitization in atopic compared to non-atopic patients were ammoniated mercury, benzoyl peroxide, amalgam and Bisphenol A Dimethacrylate (BIS-GMA). Conclusion: The atopic condition is not related to a higher frequency of delayed sensitization to a battery of dental materials.

** Key words:** Patch test, delayed-type sensitization, allergy contact, atopia, dental materials.

## Introduction

The materials used in odontology have diverse origins and natures, including antiseptics, metals, alloys, porcelains, impression materials, local anesthetics, cements, latex gloves, rubber dams, acrylates, adhesives, mouthwashes, and others (1-5). Kanerva et al. (6) identified more than 130 possible allergens derived from materials for use in odontology. Hypersensitivity reactions to dental materials are rare (3,4). The likely causes of this could be: 1) the presence of saliva in the mouth, which creates drag, dilutes and eliminates allergens; 2) the presence of keratinization in some areas of the mucosa, which impedes the binding of hap-tens; 3) the high tissue vascularization makes it capable of eliminating allergenic molecules from the area; 4) the oral mucosa has a marked mechanical resistance and 5) the low cellular density of Langerhans cells as compared to skin. This also explains the greater prevalence of hypersensitive reactions on the skin than the mucosa. (3,7-9). Diverse studies have attempted to establish a relationship between the atopic condition and development of contact hypersensitivity reactions. On one hand, some authors have pointed out that contact allergic reactions are less frequent in atopic patients than in non-atopic patients (10). Others suggest that the atopic condition represents a greater disposition to develop contact hypersensitivity (11) or that it is associated with a higher frequency of sensitization as determined with patch tests, compared to non-atopic patients (12). On the other hand, some authors found no relationship between atopia and contact hypersensitivity using IgE measurements and Prick tests as a marker of this condition (13,14). However, the relationship between atopia and delayed sensitization has not been studied for dental materials. The objective of this study was to determine if the atopic condition is related to a greater susceptibility to develop delayed sensitization to dental materials.

## Patient and Methods

 -Patients

The sample was composed of 40 atopic subjects and 40 non-atopic subjects, of both sexes, between 20 and 65 years of age. Atopic patients were recruited from the Allergy Center of the Clinical Hospital and non atopic subjects were selected from patients that requested dental treatment at the School of Dentistry of the University of Chile. All of the selected patients signed an informed consent agreement. The study protocol was approved by the ethics committees of the participating institutions and has been conducted in accordance with the ethical principles included in the World Medical Association Declaration of Helsinki. The participants were considered atopic when at least two of the following criteria were present: having a clinical history of atopic dermatitis, allergic rhinitis, allergic asthma, or food allergies; each confirmed by prick test. The non-atopic patients were defined as those that did not present signs or symptoms of immediate allergies at the taking of the history, a situation confirmed by application of the ISAAC survey (15). None of the patients were taking any drugs as topical and systemic steroids or antihistamine medications during the last month following the Allergy Center of the Clinical Hospital protocol.

 -Patch testing

**Figure 1:**
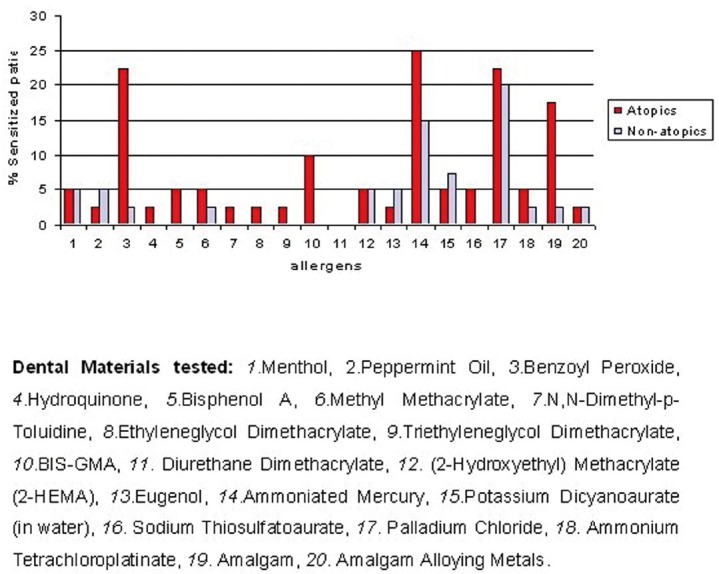
Comparison of frequency of sensitization per allergen in atopics and nonatopics patients.

The determination of delayed sensitization to dental materials was performed by patch test (25x10 Finn Chambers), which included a negative control (Vaseline) plus 20 allergens (Fig. 1) (Hermal Trolab® Dental Materials set, Germany). This allergen set was revised and produced according to the recommendations of the International Contact Dermatitis Research Group (ICDRG) and the European Environmental and Contact Dermatitis Research Group (EECDRG). These were placed on the upper back away from the midline. A first reading was made at 48 hours and a second at 72 hours after applying the patch. The following was considered evidence of sensitization: erythema, infiltration (papular reaction), edema and/or vesicles (16).

 -Clinical Exam

Two oral pathologists, both dental surgeons, carried out an intra-oral clinical exam in the Dento-Maxilo-Facial Service at the University of Chile Clinical Hospital. The lesions encountered were documented photographically and diagnosis was made by clinical and anatomopathological examination. For all patients the type and location of dental restorations or prosthetic devices were registered. All the data were recorded in a case report form in duplicated and managed in a Microsoft Office Excel 2003 ® by an assistant researcher.

 -Statistical analysis

Descriptive statistical analysis was carried out based on proportions and mode. The association between atopic condition and sensitization to dental materials was carried out with chi-square tests (X2), using a significance level of p ≤ 0.05, with a confidence interval of 95% in Data Analysis and Statistical Software STATA ® 10.0.

## Results

The atopic group consisted of 40 patients, 28 (70%) women and 12 (30%) men, with an average age of 34.6 years. The non-atopic group was formed by 40 patients, 30 (75%) women and 10 (25%) men, with an average age of 41.87 years. Non-statistically significant difference was observed. Of the entire group of patients (n=80), 61.25% presented sensitization to at least one of a battery of dental materials, with palladium chloride (21.25%), ammoniated mercury (20%), benzoyl peroxide (12.5%) and amalgam (10%) being the most frequent. In terms of the intensity of sensitization, only palladium chloride provoked a “severe” response (+++) in 3.75% of the patients and a “moderate” response (++) in 3.75% of the cases. Ammoniated mercury produced “moderate” responses in 3.75% of the patients. The materials with the greatest frequency of sensitization for the group of atopic patients were: ammoniated mercury (25%), benzoyl peroxide (22.5%), palladium chloride (22.5%), amalgam (17.5%) and Bisphenol A Dimetha-crylate (BIS-GMA) (10%). 

On comparing the frequency of sensitization between atopic and non-atopic patients, 67.5% of the former presented sensitization, against 55% obtained with non-a-topic patients. However, this difference was not statistically significant (p> 0.05). The frequency of sensitization by group and by dental material tested is compared in Figure 1. Those that presented the greatest difference in sensitization for the atopic group with respect to the non-atopic group were ammoniated mercury (25% vs. 15%), benzoyl peroxide (22.45% vs. 2.5%), amalgam (17.5% vs. 2.5%) and BIS-GMA (10% vs. 0%). However, only the benzoyl peroxide difference was statistically significant (p<0.05). 

Only 3 patients presented lesions: one atopic patient presented a lichenoid lesion on the inner surface of the cheek related to an amalgam restoration. The clinical diagnosis was confirmed by a biopsy, which showed a typical histological features of lichenoid reaction (epithelium with hyperkeratosis, basal cell layer degeneration, and band-like lymphoplasmacytic infiltration below the epithelio-mesenchymal junction), and by a positive reaction to ammoniated mercury (+++) and amalgam (++) in the patch test. Two other patients, one atopic and the other nonatopic, presented desquamative type lesions on the fingertips of both hands, together with a positive patch test to methyl methacrylate (++) and (2-hydroxyethyl)- methacrylate (2-HEMA) (++) in the case of the atopic patient and to palladium chloride (++) in the case of the non-atopic patient. Both cases corresponded to occupational dermatitis, a dentist and a metal factory worker, respectively.

## Discussion

According to the data of our study, there was not difference between atopic and non-atopic condition in relation to frequency of dental materials sensitization. Other differences between the groups, like age structure or gender were not related with the frequency of sensitization. The literature mentions that atopic patients have a greater risk to develop allergic phenomena when compared to non-atopic patients. Similarly, the presence of IgE-mediated sensitization produces an increase in the susceptibility to develop hypersensitivity to allergens tested by prick test (17, 18). With respect to delayed cell-mediated allergic responses, the literature is similar. Evidence indicates that the existence of delayed-type sensitization to one or more allergenic species augments the susceptibility for the same to occur with others (for example nickel, cobalt and chrome) (18) and the more severe or intense the response in the patch test, the greater the risk to develop an allergy to these allergens or to become sensitive to others (hyper-sensitivity IV) (18-20). Nevertheless, other studies have not been able to find a relationship between these variables (13, 14). Our results did not confirm a relationship between a greater sensitization mediated by type IV responses and the atopic condition, which is mediated by a type I mechanism. Similar findings showed Raap et al. (21) about the low frequency of history of atopy among patients with positive patch test reaction to dental materials. These results likely differ from other studies due to the diverse definitions of atopia that have been employed by different authors (10-14). 

The allergens tested in this study that showed the greatest frequency of sensitization were: palladium chloride, ammoniated mercury, benzoyl peroxide and amalgam, which differed from what has been reported in some other studies (2, 16). However the high palladium sensitization is similar to others studies (21, 22) and the amalgam and mercury are the most dental material with positive patch test reaction in others (23). A possible reason for the higher sensitization is that palladium has become and important contact allergen because of increased use in industry, jewelry, and dentistry (24). The differences in frequency of sensitization could be due to the fact that each population group presents distinct, genetically determined immunological characteristics, specific allergens according to region, and differences in lifestyle, therapeutic practices and supplies used by local industries (17,25). An example of this is observed in the frequency of sensitization to allergens contained in amalgam restorations. Ammoniated mercury presented a frequency of general sensitization of 20%, a value that differs from those found in other studies, which report a maximum percentage of 13% (16,26). The percentage of sensitization to amalgam in this study was 10%, which is much greater than other studies, which report estimated values of 1.1% (16). These reports come from developed countries, mainly Scandinavian, where the use of amalgam as a restoration material is questioned, being considered toxic to the environment and to human beings. It is also known that in these countries the incidence of decays is lower with respect to Latin American countries where, in contrast, amalgam is permitted as a restoration material and is used extensively in primary healthcare centers. However in East European countries the frequency of amalgam sensitization is similar to Latin America (23), probably because the frequent use of amalgam restoration. 

In the analysis of sensitization frequency to amalgam and ammoniated mercury between the study groups, we observed that the atopic patients had a higher frequency of sensitization to these allergens than non-atopic patients. However, this difference was not statistically significant.

Of the group of acrylates, 2–HEMA (2-hydroxyethyl methacrylate) and BIS–GMA showed the greatest percentage of sensitization (5%). This value is in agreement with others found in the literature, which describes a range of 2.8% to 5.8% (16,27). Only benzoyl peroxide (BP) showed statistically significant difference between atopic and non-atopic groups (22.45% vs. 2.5%). But, we have to consider that BP was the highest dental material that provoked irritative reactions or doubtful lectures (+/-), with 27.5% in atopics and 30% in non atopics patients. This material could be difficult on visual examination to differentiate between allergic or irritative reactions. We had a score of -0.69 in the Kanerva reaction index (16); otherwise Kanerva showed a score of -0.51, this means that BP shows a tendency to induce skin reactions hardly distinguishable between allergic or irritative. However, the BP sensitization proportions, were significantly different between atopics and non atopics subjects, with values of 22.45% and 2.5%, respectively; this finding suggest that besides the irritative responses, it induce sensitization reactions in atopic patients. The origin of patient’s sensitization to this allergen may be due to other source of sensitization, i.g. wheat flour, cosmetic products, etc. 

The clinical exam detected a lesion in the oral mucosa attributable to an immunoallergic etiology in an atopic patient (lichenoid reaction). Two patients (one atopic and another non-atopic) showed immunoallergic lesions on the skin of the hands. On relating the sensitization results to the clinical expression of oral lesions of allergenic origin, no association was observed between the high percentage of patients sensitized to some material and the presence of clinical manifestations of lesions (hypersensitivity). This result is in agreement with other studies that show not oral clinical relevance for positive patch test reaction to dental materials (21). This suggests that the oral mucosa could be an important route of sensitization; however, it presents a high grade of tolerance. Considering that the oral mucosa is part of the epithelial barrier of the digestive system, the need for tolerance to an extensive amount of antigens is apparent. Of 23 patients that had contact dermatitis due to metals and had restorations of this type in the mouth, none demonstrated the presence of lesions during intra-oral examination. Contact dermatitis to metals does not necessarily imply that an allergic contact mucositis to metals must result when these are in contact with the oral mucosa. 

Through the use of the Prick test and the clinical history of allergies to establish a diagnosis of atopia, it was observed that patients with this condition would not present more susceptibility to develop delayed-type sensitization to dental materials commonly used in clinical practice, i.e. the atopic condition would not be related to a higher frequency of sensitization to a battery of dental materials.
